# Vaccine value profile for schistosomiasis

**DOI:** 10.1016/j.vaccine.2024.05.068

**Published:** 2024-07-23

**Authors:** Gavin Yamey, Kaci Kennedy McDade, Roy M. Anderson, Sarah M. Bartsch, Maria Elena Bottazzi, David Diemert, Peter J. Hotez, Bruce Y. Lee, Donald McManus, Adebayo J. Molehin, Meta Roestenberg, David Rollinson, Afzal A. Siddiqui, Miriam Tendler, Joanne P. Webster, Hong You, Raphaël M. Zellweger, Caroline Marshall

**Affiliations:** a Duke Global Health Institute, Duke University, Durham, NC, United States; b Faculty of Medicine, School of Public Health, Imperial College London, London, United Kingdom; c Public Health Informatics, Computational, and Operations Research and Center for Advanced Technology and Communication in Health, City University of New York Graduate School of Public Health and Health Policy, New York City, NY, United States; d National School of Tropical Medicine, Baylor College of Medicine, Texas Children’s Hospital Center for Vaccine Development, Houston, TX, United States; e School of Medicine and Health Sciences, George Washington University, Washington, D.C., United States; f QIMR Berghofer Medical Research Institute, Brisbane, Queensland, Australia; g Department of Microbiology & Immunology, Midwestern University, Glendale, AZ, United States; h Leiden University Medical Centre, Leiden, Netherlands; i Global Schistosomiasis Alliance, London, United Kingdom; j Center for Tropical Medicine & Infectious Diseases, Texas Tech University Health Sciences Center School of Medicine, Lubbock, TX, United States; k Laboratory of Research and Development of Anti-Helminth Vaccines, Oswaldo Cruz Institute (Fiocruz), Rio de Janeiro, Brazil; l Department of Pathobiology and Population Sciences, Royal Veterinary College, University of London, United Kingdom; m International Vaccine Institute, Seoul, Korea; n World Health Organization, Geneva, Switzerland

**Keywords:** Vaccine value, Schistosomiasis, Neglected tropical diseases, Parasitic infection

## Abstract

Schistosomiasis is caused by parasitic flatworms (*Schistosoma*). The disease in humans can be caused by seven different species of *Schistosoma*: *S. mansoni*, *S. japonicum, S. haematobium, S. malayensis, S. mekongi, S. guineensis* and *S. intercalatum*, as well as by hybrids between species, including livestock schistosome species. People are infected when exposed to infested water and the parasite larvae penetrate the skin. Poor and rural communities are typically the most affected, and the general population who lives in affected areas and is exposed to contaminated water is at risk. Areas with poor access to safe water and adequate sanitation are also at heightened risk. About 236.6 million people required treatment for schistosomiasis in 2019—mostly people living in poor, rural communities, especially fishing and agricultural communities.

This ‘Vaccine Value Profile’ (VVP) for schistosomiasis is intended to provide a high-level, holistic assessment of the information and data that are currently available to inform the potential public health, economic, and societal value of pipeline vaccines and vaccine-like products. This VVP was developed by a working group of subject matter experts from academia, non-profit organizations, public private partnerships, and multi-lateral organizations. All contributors have extensive expertise on various elements of the schistosomiasis VVP and collectively aimed to identify current research and knowledge gaps. The VVP was developed using only existing and publicly available information.

## 1. The global public health need for a vaccine

Schistosomiasis is caused by parasitic flatworms (*Schistosoma*) [1]. The disease in humans can be caused by seven different species of *S. mansoni*, *S. japonicum*, *S. haematobium*, *S. malayensis*, *S. mekongi*, *S. guineensis* and *S. intercalatum*, as well as by hybrids between species, including livestock schistosome species. The first three species account for the current magnitude of human schistosomiasis globally. People are infected when exposed to infested water and the parasite larvae (cercariae) penetrate the skin [2]. Freshwater snails are the intermediate hosts [3]. Infected humans contaminate water sources through their excretions (feces or urine), which contain eggs which hatch into the second larval stage (miracidia) [2].

The general population who lives in affected areas and is exposed to contaminated water is at risk [3]. Areas with limited access to safe water and adequate sanitation are at heightened risk [4]. About 236.6 million people required treatment for schistosomiasis in 2019—mostly people living in poor, rural communities [2], especially fishing and agricultural communities [5] (Table 1).

### 1.1. Current methods of surveillance, diagnosis, prevention, and treatment

Parasite egg detection has been the central method for evaluating schistosomiasis control programmes. It has relied upon the Kato Katz method for detecting eggs in faeces and by urine filtration for eggs in urine. Counting excreted eggs can provide data relating to prevalence and intensity of infection but these traditional methods lack the required sensitivity especially in low prevalence and intensity settings. New diagnostic methods based on circulating cathodic antigen (CCA) and circulating anodic antigen (CAA) have been implemented and are being further developed together with novel molecular approaches. WHO recently published the Diagnostic Target Product Profiles for monitoring, evaluation and surveillance of schistosomiasis control programmes [47].

In support of the WHO NTD Roadmap for Neglected Tropical Diseases, WHO published new guidelines in 2022 for the control and elimination of schistosomiasis, which advocate for an integrated approach [48]. Treatment of the disease relies on the drug praziquantel given orally as a single dose of 40 mg/kg primarily through mass drug administration (MDA) programmes. Currently, Merck KGaA donates up to 250 million tablets on an annual basis, which is roughly sufficient to treat 100 million school-age children. The new guidelines recommend annual preventive chemotherapy in endemic communities with a prevalence of 10% or above for all age groups, beginning from two years old. In communities where infection levels remain high, countries are encouraged to consider biannual treatments and in communities with a prevalence below 10% annual preventive chemotherapy should continue or a test and treat approach should be developed. The recognised need to treat adults within the community, given that emphasis was previously on treating school-aged children, raises an important question as to whether the likely increased demand for praziquantel can be met by current global supplies. Monitoring and evaluation programmes are essential to assess the impact of programmes to help improve efficiency. Most prevalence and intensity data have been collected from school-aged children and there is a need to collect data from a broader age-range to include adults [48,49].

To eliminate schistosomiasis, a cross cutting approach will be needed to reduce transmission. Such an approach will require additional inputs in areas such as water, sanitation, and hygiene (WASH); education and behaviour change; integrated snail control; appropriate veterinary and animal health measures to guard against zoonotic infections; and increased capacity in the health system, especially at the level of the health centre. The International Task Force for Disease Eradication in its overview of schistosomiasis and the new WHO guidelines encouraged further research and development of schistosomiasis vaccines, as a vaccine may be crucial for eliminating schistosomiasis as a public health problem [50].

### 1.2. Summary of knowledge and research gaps in epidemiology, potential indirect public health impact and economic burden

Prior to wide-spread MDA, many extensive long-term studies have documented the epidemiology of the major human schistosome infections [51]. The main epidemiological features are convex age intensity and prevalence curves, with the declines in older age groups due to some combination of exposure and acquired immunity. Fecundity of adult female worms is density dependent since the per worm fecundity declines as worm burden rises as demonstrated by detailed autopsy studies to assess worm load and egg output [52]. Adult worm life expectancy is uncertain but thought to be around 3–5 years on average. It is thus longer than the soil transmitted helminths, but shorter than the human filarial worms. Bounce back time post cessation of chemotherapy is of the order of a few years to around 90% of per MDA levels. This is a much slower bounce back time when compared with soil transmitted helminths, but shorter than the filarial worms. The largest uncertainty in our current epidemiological understanding of these infections is what determines the convex age intensity of infection curves (immunology or ecology/human behaviour). This uncertainty is highly pertinent to vaccine development since if natural infection only builds partial immunity slowly after repeated exposure as individuals age, then vaccines will have to do better than the human immune response to natural infection. Human helminths have large genomes (often as much as 1/3 the size of the human genome) so it is possible that they make many secretions to modulate immunological attack by the human host.

## 2. Potential target populations and delivery strategies

Historically, schistosomiasis control has relied heavily on MDA, mainly focusing on school-aged children. With a recent public health paradigm shift from schistosomiasis control to elimination, expanding the scope from school-aged children to all age-groups (including pre-school age children and adults) and particular risk groups (such as fishermen, farmers of irrigation fields and car washers) seems appropriate and is supported by mathematical modelling studies of MDA impact [53]. Similarly, vaccine roll-out could start with school-based programs for practical reasons, or indeed, as a part of current infant vaccination programmes at around 2 years of age that target the common viral infections such as measles, and subsequently be expanded to the wider community, including other age and risk groups. This could ultimately result in a combined, infant, school- and community-based delivery strategy, which has recently been shown to generate high coverage levels [54].

To maximize impact on disease burden, schistosomiasis vaccination could be used in conjunction with other interventions such as MDA, and water, sanitation, and hygiene (WASH) improvements [3,55]. However, the desirability of this will depend greatly on vaccine efficacy and the duration of protection it provides to an individual. If highly efficacious and of long protection duration, vaccination alone could be the most effective intervention. In high-endemicity regions, vaccine delivery should be integrated with the existing health system to maximize possible synergies [56]. This integrated approach may result in a delivery strategy combined with other public health activities such as: (i) routine infant measles or childhood vaccination for pre-school children; (ii) regular MDA activities for school age children; and/or (iii) HPV vaccination for girls, boys, and women. In addition, ad-hoc campaigns will be tailored to particular target populations or high endemicity regions. Ideally, initiation of large-scale vaccination campaigns in endemic areas should follow MDA, with the aim of reducing re-infection, as above. With vaccine coverage above 70% of the total population, vaccination with appropriately safe and immunogenic vaccines should be introduced into the childhood immunization calendar in endemic countries starting at 12 months of age.

Of note, should age of MDA expand to lower age groups, thanks to upcoming availability of paediatric praziquantel orally dispersible formulation [12], vaccine delivery could be concomitantly expanded to these same lower age groups or integrated with infant vaccination programmes. The definition of age range for vaccination needs to be adequate to the safety and immunogenicity characteristics of licensed vaccines (Table 2).

## 3. Schistosomiasis and its consideration as a public health priority by global, regional or country stakeholders

There are many international stakeholders, non-governmental organizations, and government ministries involved in schistosomiasis control and elimination in Africa that, potentially, would have significant or prioritized interests in a vaccine for schistosomiasis.

There is interest from funders globally to reduce the impact of schistosomiasis, as evidenced by ongoing efforts using various approaches such as chemotherapy of at-risk groups, access to clean drinking water and improved sanitation, hygiene education, environmental management, and snail control. Praziquantel, which is used for chemotherapy, is donated by Merck (250 million tablets a year).

In areas where zoonotic schistosome species occur, including *S. japonicum* and *S. mekongi* in Southeast Asia, as well as is now acknowledged across Africa too, veterinary and animal health measures will be required, and vaccines are a potential intervention strategy. This may be particulary pertinent given recent reports of systematic mis-use of praziquantel intended for humans and/or incorrect dosage veterinary formula praziquantel being given by subsistence farmers to their African domestic livestock, thereby also potentially facilitating the emergence of praziquantel resistance [33,46,58].

Transmission blocking vaccines that prevent infection or reduce fecundity of adult worms have shown some level of protective efficacy against *S. japonicum* in bovines in the field [59,60]. Furthermore, social surveys amongst African subsistence farmers have demonstrated both a need and a demand, including a willingness to pay, for measures aimed at preventing livestock schistosomiasis [33,46,58]]. Effective livestock vaccines could also help reduce access and evolutionary pressures on praziquantel.

WHO launched a NTD Road Map for 2021–2030 that targets the elimination of schistosomiasis as a public health problem in all endemic countries [6]. The schistosomiasis annex to the Road Map lists as a required action: “consider development of a vaccine for humans and animals to prevent reinfection and reduce transmission.”

The new WHO Guideline on Control and Elimination of Human Schistosomiasis puts forward six recommendations using currently available tools, hence this guideline does not include the use of schistosomiasis vaccines, since they are not yet available [61].

In 2020 estimates suggest that at least 241.3 million people, the vast majority in sub-Saharan Africa, required treatment for schistosomiasis. The potential market for a vaccine, in terms of number of potential doses required in each geographical area, should be determined. The market will depend on the target group, taking into account the product profile of the vaccine or vaccines (likely efficacy against the various species of *Schistosoma*, safety in different ages, vaccination schedule/number of doses, shelf life/storage/transport, etc.).

Because of the known distribution of schistosomiasis, any vaccine would be unlikely to be a ‘dual market’ vaccine (with potential demand and uptake in both HICs and LMICs). However, a private market for travellers from HICs to affected areas in LMICs is possible (but would be relatively small) alongside a vaccine for military personnel (Table 3).

## 4. Existing guidance on preferences/preferred product attributes for vaccines against schistosomiasis

Candidate vaccine agnostic guidance on preferred product attributes for schistosomiasis have been developed by the National Institute of Allergy and Infectious Diseases. This information is presented in Table 4. There are two key properties of any vaccine. The first is vaccine efficacy, determined as a percentage of those immunised who are protected against infection, or a more complex definition of efficacy based on the fraction of worms suppressed by vaccination from developing to mature adults or the fractional reduction in eggs. Second, and of equal importance, is the duration of protection—which is particularly important when compared with MDA. Understanding duration of protection requires long-term phase III trials or phase IV studies.

## 5. Vaccine development

### 5.1. Probability of technical and regulatory success (PTRS)

Vaccine development for schistosomiasis includes the development of both human and veterinary vaccine candidates, as the implementation of an animal-based transmission-blocking vaccine may present an important part of a package of integrated control measures synergizing human and animal health.

Preferential targets for vaccine development are *S. mansoni* and *S. haematobium*, the main species with impact in endemic countries, especially in Africa as the etiologic agent of urinary tract and female genital schistosomiasis. Products from different manufacturers should be preferentially interchangeable.

#### Human vaccines for intestinal schistosomiasis caused by *Schistosoma mansoni* and urogenital schistosomiasis caused by *Schistosoma haematobium*

**Fig. 1.**
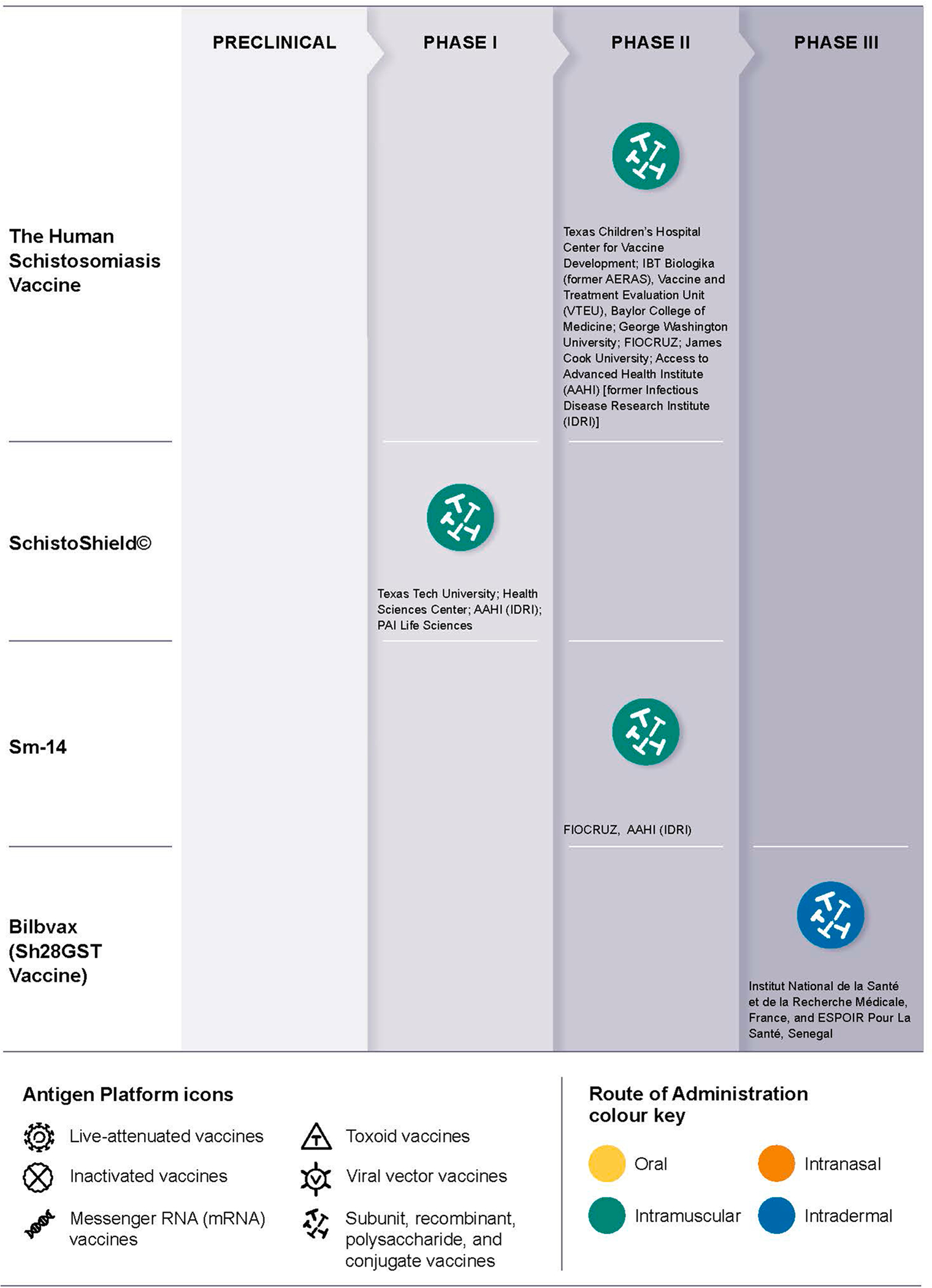
Overview of vaccine candidate in clinical trials.

Proof-of-concept for vaccinating against schistosomiasis accelerated in the 1970s and 80s through an improved understanding of the mechanisms underlying protective immunity to *S. mansoni* and *S. haematobium* infections in animal models [73–75], followed by efforts to elicit protection against experimental challenge infection in these models using irradiated cercariae or schistosomulae. These early animal vaccine studies were conducted in both the U.S. at the Biomedical Research Institute in Rockville, Maryland [76,77], and in the U.K. at the London School of Hygiene and Tropical Medicine [78]. Previously, in the 1960s, S.Y. Li Hsu and H.F. Hsu at the University of Iowa demonstrated protective immunity in Rhesus monkeys with infectious, non-irradiated cercariae of *Schistosoma japonicum* against subsequent experimental challenge infection [79] Following efforts to reproduce this protective immunity by substituting homogenates or extracts of cercariae or schistosomulae [80]. The next advance was to test individual surface antigens isolated by affinity chromatography [81]. Later, during the mid-1980s, the first schistosome antigens were expressed as recombinant proteins [82]. Over the ensuing decade, multiple internal, surface, and excreted recombinant schistosome antigens underwent animal testing, although six of the promising candidates that were selected by an expert review group convened during the 1990s by the World Health Organization and the Special Programme for Research and Training in Tropical Diseases, failed to achieve high levels of protection in independently conducted mouse challenge trials. However, the laboratory of R.A. Wilson at the University of York has since identified shortcomings in the mouse model that limit its utility for adequately estimating protective immunity of candidate vaccines for human schistosomiasis [83,84]. For an animal model, the baboon, a natural host of schistosomiasis appears to be an excellent model to evaluate safety, immunogenicity and protective immune responses because these animals exhibit human-like acute and chronic disease manifestations [85]. Now, as shown in Fig. 1 and Table 6, newer recombinant proteins have undergone more refined testing in animal models with the benefit of newer and potentially more effective adjuvants, together with human immunology studies; this newer generation of recombinant protein schistosome vaccines have entered into Phase 1–2 clinical testing in the U.S., Brazil, and Africa.

#### Veterinary vaccine for bovines (water buffalo/cattle) in Asia to reduce/eliminate human infection by *Schistosoma japonicum*

The schistosomiasis annex to the WHO Road Map lists as a required action: “consider development of a vaccine for humans and animals to prevent reinfection and reduce transmission.”[6]

Schistosomiasis control in the Philippines and China is complicated by the zoonotic nature of the disease with bovines (water buffalos and cattle), which act as major reservoir hosts for *S. japonicum,* being responsible for, depending on the habitat type, up to 90% of environmental contamination of the parasite’s eggs [86]. However, in terms of vaccine development, this zoonotic nature permits a step-wise approach that would start with the use of a “transmission-blocking” veterinary vaccine for bovines before moving on to a product aimed at human vaccination, should this be necessary [34]. Accordingly, vaccination of animals, particularly bovines, is considered useful with respect to schistosomiasis japonica, and should follow human vaccination as a complementary tool. The vaccine would assist in long-term prevention of human (and animal) infection, a concept supported by mathematical modelling, which predicts that a schistosome vaccine capable of reducing the faecal egg output in bovines by 45% in conjunction with praziquantel treatment would lead to a significant reduction in the transmission of schistosomiasis japonica almost to the point of elimination [87].Similar step-wise approaches could plausible be of value across sub Saharan Africa too, particularly where targeting ongoing zoonotic transmission of haematobium group schistosomes from livestock to humans.

Vaccination can reduce egg excretion from cattle and buffalo, thereby interrupting transmission from bovines to snails. The use of such a vaccine would be particularly applicable in areas deemed unsuitable for the replacement of bovines by mechanized farming, one of the interventions featuring in the current integrated schistosomiasis control strategy for China [88]. Indeed, the implementation of an animal-based transmission-blocking vaccine as part of a package of integrated control measures sits well with the One Health concept synergizing human and animal health. This was a conclusion reached at two workshops, co-sponsored by the National Institute of Allergy and Infectious Diseases (NIAID) and the Bill and Melinda Gates Foundation, on schistosomiasis elimination strategies and the potential role of a vaccine for Asia [55,57]. Discussion at one of these workshops on the question of a clinical versus a veterinary vaccine concluded the latter was considered as the preferred target product profile (TPP) with respect to schistosomiasis japonica as the less rigorous safety concerns required would allow more rapid implementation [57]. A large number of *S. japonicum* transmission-blocking vaccine candidates have been identified with Sj-GST26, Sj-GST28, Sj-97 (paramyosin), Sj-TPI (triose-phosphate isomerase) and Sj-IRs (insulin receptors) the most tested [55,57,89,90]. In addition, the Fatty Acid Binding Protein family member Sm14, which shares homologous members with the main schistosome species and other helminths of human and veterinary importance, is being developed as an anti-Fasciola vaccine for livestock (Livac). Safety and immunogenicity were demonstrated in definitive hosts such as sheep and cattle in Brazil by Fiocruz and Ourofino Animal Health groups, and goats by the Spanish group from Cordoba University, among others.

The development of a vaccine for schistosomiasis japonica has proven highly challenging but the deployment of a transmission blocking vaccine, in tandem with other interventions (e.g., improved water, sanitation and hygiene, health education and snail control) for the prevention of *S. japonicum,* would be invaluable if the goal of elimination is to be achieved.

#### Veterinary vaccine for livestock and wildlife in Africa to reduce/eliminate human infection

The role of livestock in the transmission of schistosomiasis in Africa and beyond, namely by *S. haematobium* group parasites, has now been acknowledge by WHO, FAO and beyond, largely demonstrated through high prevalence and intensity levels of the human *S. haematobium* with livestock *S. bovis, S. curassoni*, or *S. mattheii* [5,33], as well as cases of human infection with livestock-only hybrids of *S. bovis* with *S. curassoni* [91]. Furthermore, whilst zoonotic spillover to human infection through recent hybridization may be currently low, it has been predicted to become more important as elimination programmes continue [9]. Therefore, in areas where potential zoonotic transmission occurs, the need for development of livestock vaccines for use within Africa should be raised too. As mentioned earlier, this may be particularly relevant given recent reports of mis-use of praziquantel intended for humans and/or incorrect dosage veterinary formula praziquantel being given by subsistence farmers to their African domestic livestock [33,46,58]. Furthermore, social surveys amongst African subsistence farmers have demonstrated both a need (largely in terms of reducing the mortality and morbidity, and thereby financial losses, of their infected herds) and a willingness to pay for measures aimed at preventing livestock schistosomiasis [33,46,58]]. Finally, there may also be a role for the *S. mansoni* vaccines mentioned above for wildlife as well—notably, for the non-human primates who we know also perpetuate transmission and key challenges for areas to achieve verification of interruption of transmission status.

### 5.2. Overview of the vaccine candidates in the clinical pipeline

#### Controlled human infection models for advancing vaccine development

Controlled human infection models (CHIMs) are valuable tools to advance vaccine and drug development and can be used to efficiently test new vaccine or drug candidates. Moreover, controlled human infections can provide insights into immunological responses that could help identify correlates of protection or new targets. In these ethically contentious studies, a small number of healthy volunteers are deliberately exposed to a pathogen, after which they are followed over time before the infection is cleared, often by curative treatment. Particularly in the field of malaria, controlled human infections have helped advance vaccine candidates such as the RTS,S malaria vaccine. The initial efficacy of RTS,S was shown in a proof-of-concept CHIM study, and later confirmed in a phase 3 clinical trial in African children (which showed about 30% protective vaccine efficacy) [96].Analogous to malaria, a CHIM has been developed using *S. mansoni* cercariae. To ensure safety for volunteers, a single-sex infection model was developed, whereby healthy volunteers are exposed to cercariae of one gender only. This will ensure that worms are not able to mate and produce any eggs, which are the main cause of morbidity.

In the first male-only controlled infection study with *S. mansoni* (Sm), challenge with 20 male cercariae was well-tolerated and led to detectable infection in 82% (9 out of 11 volunteers) based on serum circulating anodic antigen levels [106]. Interestingly, some volunteers develop systemic symptoms at weeks 4–5 after exposure, which are reminiscent of acute schistosomiasis syndrome (Katayama fever), whilst eosinophilia occurs somewhat later. Rather, initial immune responses were characterised as mixed Th1/2 cellular responses with high levels of IgG1 induced. No eggs were detected in stool of these volunteers at any timepoint, confirming the single-sex approach.

More recently, a study using female cercariae was initiated [107] to investigate whether this model could add to the toolbox for schistosomiasis vaccine development. For both human models, the fact that they use single sex schistosomes ensures safety to participants but also is an important difference with field infections. Infection levels, as measured by circulating antigens, are manyfold lower as compared to those found in highly endemic areas, for example in Sub-Saharan Africa. The healthy volunteers included in controlled infection studies differ from any vaccine target population with regards to genetics, infection history and very often age. In order to bridge the gap between infection models and endemic infections, a controlled infection model in endemic areas could be insightful. Currently, efforts are underway to also establish these technically advanced models in Uganda. Furthermore, comparison of (vaccine) immune responses between non-endemic and endemic populations will further our understanding of schistosomiasis and aid vaccine development.

Lastly, a third study is ongoing where healthy volunteers are repeatedly exposed to Sm male cercariae, in order to explore whether the early mixed Th1/Th2 inflammatory responses that seem to be associated with systemic symptoms are a sign of natural protection or will ultimately lead to tolerogenic response [108]. Such exploratory studies provide a unique opportunity to identify correlates of protection that improve our understanding of immunity and that may lead to the discovery of new vaccine targets.

## 6. Health impact of a vaccine on burden of disease and transmission

The value of a schistosomiasis vaccine depends on its ability to prevent disease and/or reduce the burden of disease and its sequelae (e. g., intensity level of infection, chronic disease, death) – and, most importantly the duration of any such effects. Thus, it is important to have a sense of what is already known about the health burden of schistosomiasis and its transmission dynamics to understand what a vaccine should look like in terms of reducing these things. Such information can be used to develop a ‘wish-list’ of characteristics, features, and attributes the vaccine should have once it reaches the market (i.e., a target product profile, TPP) [109]. Understanding a vaccine’s value and developing this list prior to licensure can help guide vaccine development while still time to make adjustments [109]. Since both schistosomiasis and vaccines reside in and are part of complex systems, mathematical and computational models that represent these systems can help inform TPPs by helping decision makers (e.g., vaccine developers, funders, manufacturers, policy makers) understand and address complex systems. For example, models can help identify the efficacy thresholds at which things change, which can help set efficacy targets for which vaccine developers can aim. These models can help save time, effort, and resources, often associated with clinical studies, and they can help show other impacts/effects that may not otherwise have been seen as well as the future/long-term impacts, both of which are hard to see and measure in traditional clinical studies. Therefore, Table 7 summarizes modelling studies that explore the use of a schistosomiasis vaccine and quantify its potential impact on schistosomiasis’ disease burden and/or transmission.

### 6.1. Summary of knowledge and research gaps in modelling health impact on disease burden and transmission

#### Priority Knowledge

- Most studies that evaluate vaccination look at achieving the WHO morbidity and elimination goals (i.e., a prevalence of heavy intensity infection in school-aged children ages 5–14 of ≤5% and ≤1%, respectively) and use interrupting community transmission as the outcome metric.
- Vaccination alone seems to be able to achieve the WHO goals, but would take several years to achieve, especially in high transmission settings. The predictions depend critically on vaccine efficacy and duration of protection.
- Studies indicate that vaccination used in combination with MDA would tend to provide greater benefits (e.g., the combination would reduce transmission more quickly) than using either alone. But vaccination alone would work well if vaccine efficacy was high and the duration of protection was long.
- Interrupting transmission (i.e., achieving WHO goals) is more readily achieved in low transmission settings compared to moderate and high transmission settings, but again this depends on vaccine efficacy and duration of protection.
- The duration of protection seems to be the largest driver of vaccination’s impact across all the studies. For example, if protection lasts less than 5 years, there is very little impact as repeated vaccination is needed. Studies seems to indicate that 5–10 years duration of protection or more is ideal.
- Mass vaccination of the community should more quickly interrupt transmission and has the greatest chances of achieving the WHO goals compared to vaccinating other target populations.
- Vaccinating school-aged children seems to be the next best strategy, followed by infant vaccination.

#### Research Gaps

- Future studies may include other outcomes of interest e.g., prevalence in humans as referenced by the mean worm burden; snail infection levels are always low − even in high human prevalence settings − and are not a reliable marker of impact when compared with intensity measures in humans. Most of the current studies focus on interrupting transmission but may miss other benefits of vaccination (e.g., reductions in prevalence, heavy intensity infections, reductions in mean worm burden).
- Future studies may comprise additional regions/countries/contexts. Current studies are calibrated to Kenya and Senegal, and in the future, it is conceivable to vary conditions so as to represent other transmission settings. However, this may not adequately reflect differences between regions (e.g., baseline prevalence, population size and age structure, differences in contact with reservoir/water) that may impact the value of vaccination.
- Many of the current studies evaluate very specific scenarios. Future studies may want to identify the relationship between different vaccine characteristics (e.g., efficacy, duration) and health outcomes and reductions in transmission.

## 7. Social and/or economic impact of a vaccine

Ultimately, it is important to know the economic value of the schistosomiasis vaccine because a range of different decision makers will be using economic measures to determine where the vaccine will fall on their priority list and what resources may be needed [109,116]. For example, funders need to know where to invest limited resources and how much they should invest. As another example, vaccine developers need to understand the thresholds at which the vaccine becomes cost saving and cost-effective to set targets for various vaccine characteristics (e.g., efficacy, duration of protection, price). As another example, policy makers need to understand the trade-offs between vaccination costs and its effects (e.g., its ‘bang for the buck’) to know if a vaccine is worth developing and implementing based on costs [117]. Policy makers also need to understand what vaccination implementation characteristics are necessary (e.g., target population, coverage, frequency) to develop effective policies and programs. It is helpful to determine the economic value prior to licensure or as early in development as possible, while there is still time to make adjustments [109].

While some of the immediate effects of a schistosomiasis vaccine would be to prevent cases and/or negative health outcomes and their associated medical costs, the potential value of a vaccine extends way beyond this. A vaccine may impact other negative and longer-term consequences of schistosomiasis infections such as life-long disability, reduced economic productivity, impaired child health and development (e.g., stunting) and their ability to learn, and reduced worker productivity [118]. These negative consequences can have a larger impact on a society as a whole, especially since NTDs tend to affect the poorest and most marginalized populations and may, in fact, contribute to these populations’ poverty [117]. Thus, vaccination may prevent productivity losses, increase health benefits, reduce disability, and provide other community benefits. Therefore, economic evaluations of a schistosomiasis vaccine should include the broader impacts of a vaccine in a population to capture the whole spectrum of benefits. Table 8 summarizes modelling studies that evaluate the impact of a schistosomiasis vaccine on the economic, health, and social burden of schistosomiasis.

### 7.1. Summary of knowledge and research gaps in modelling studies that measure anticipated socio-economic impact of the vaccine

#### Priority Knowledge

- Vaccination (with an efficacy ≥ 90%) would be cost-effective (measured as cost per high intensity infection years averted) and, in some circumstances, could generate cost savings, compared to MDA from the healthcare provider perspective (including only the cost of the intervention and delivery).
- Vaccination would be more cost-effective than MDA, when the vaccine costs up to $9.20. However, this threshold cost varied by study and transmission setting (e.g., higher costing vaccines remained cost-effective in higher transmission settings) and ranged from $3 to $9.20.
- The duration of protection offered by vaccine is a large driver of its cost-effectiveness. It also is a driver of which target population it is most cost-effective to vaccinate (e.g., with a shorter duration of protection it is more cost-effective to vaccinate school-aged children).
- Transmission setting is also a driver of the vaccine’s cost-effectiveness. Vaccination would be more cost-effective in higher transmission settings compared to lower transmission settings.
- In Brazil, schistosomiasis infections are estimated to cost $20.1 million for hospitalized cases and $40.7 million for all infections.
- Cost of illness studies estimate that over 95% of total costs associated with schistosomiasis are due to productivity losses (e.g., lost wages due to illness and death).

#### Research Gaps

- There is a lack of estimates of the disability associated with schistosomiasis, with little quantification of the number of disability-adjusted life years (DALYs). A quick calculation suggests 13–15 million DALYs lost worldwide in 2004 [122]. However, updated estimates would be helpful.
- Future studies may want to evaluate the cost-effectiveness of lower efficacy vaccines (e.g., lower than 90%).
- Current studies evaluating the cost-effectiveness of vaccination do not appear to include direct medical costs. Therefore, these studies may underestimate the true value of the vaccine as they do not capture all the benefits of vaccination (e.g., reductions in hospitalization and treatment costs).
- Current studies evaluating the cost-effectiveness of vaccination do not include productivity losses (e.g., reductions in productivity due to illness and death), which may underestimate the value of vaccination. Future studies may want to capture such losses.
- Future studies may want to quantify some of the broader social impacts of vaccination (e.g., impact on learning/education and development/growth), which do not appear to have been captured by the studies appeared to date.
- Many of the current studies are calibrated to Kenya, and to a lesser extent Senegal, and the costs included are not context/location specific. Future studies will hopefully quantify the value in other regions/countries/contexts given differences that may impact the value of the vaccine (e.g., population size, age structure, contact with water/reservoir, costs, healthcare system differences).

## 8. Policy considerations and financing

Fifty-one countries require use of preventative chemotherapy treatment. Most of these countries (76%) are eligible for funding from Gavi, the Vaccine Alliance (Gavi), 84% are low or lower-middle income, and 80% are located in Africa.

A WHO policy recommendation is a pre-requisite for WHO prequalification. It is also a pre-requisite for financing by Gavi. Should a schistosomiasis vaccine become available, Gavi may evaluate it through its Vaccine Investment Strategy (VIS). The VIS process uses a robust and transparent mechanism to evaluate vaccine products based on several criteria, such as health and economic impact, contribution of equity and social protection, feasibility, and implementation costs.

Vaccine policy development and decision-making may also be influenced by the following considerations:

- For the target population of infants and young children, it may be beneficial to administer the vaccine within the infant EPI programme for optimal cost-effectiveness and feasibility of implementation, and/or to administer the vaccine alongside anthelminthic MDA.
- Rather than national roll-out, some countries may elect strategic, targeted use of schistosomiasis vaccines, especially for high-incidence areas.
- National decision-making bodies may require evidence of vaccine safety and efficacy in local populations before considering their use, irrespective of whether other countries have licensed the vaccine(s).
- Local manufacturing may greatly enhance the likelihood of government uptake, particularly where countries have national policies on self-reliance/domestic production of vaccines.
- If a schistosomiasis vaccine can be combined (co-formulated) with another vaccine (e.g., hookworm) that has a compatible delivery strategy and schedule, this will significantly impact its feasibility of delivery and cost-effectiveness.
- Table 9 outlines some of the criteria that are likely to be important for policy and financing decision making, based on published discussions with policy stakeholders, the WHO SAGE E2R framework, principles and considerations for adding a vaccine to a national immunization programme, and Gavi’s VIS criteria. In studies on school children and infants, nutritional status should be evaluated, and corresponding nutrition measures should be implemented for malnourished children/infants.

In addition to Gavi, there are other health agencies, especially regional ones, that would probably play a critical role in supporting the rollout of schistosomiasis vaccines, e.g., Africa CDC, the African Vaccine Acquisition Trust, the Asia Pacific Vaccine Access Facility, and the regional development banks.

## 9. Access and implementation feasibility

- Possibility of implementation within existing delivery systems: **High**. Vaccines in current development have relatively simple dosing schedules (see Table 6). Possibility for administration alongside MDA programs.
- Commercial attractiveness: **Moderate/High**. There is significant potential LMIC vaccine demand, and many endemic countries are eligible for Gavi support (see Table 5). There is also some HIC utility for military populations.
- Clarity of licensure and policy decision pathway: **High**; The existing standard licensure pathways are applicable, and there is an additional possibility of using CHIM data.
- Expected financing mechanism: **Moderate**. Interest from Gavi with respect to funding has not been formally confirmed, but it is expected to fall within Gavi consideration.
- Ease of uptake: **High**. There is a good existing level of acceptance of vaccination, mass drug administration, and other health care approaches, and the target populations are well defined. Country-specific commitments to vaccine introduction have not yet been determined.

## 10. Conclusion

The global public health need for a schistosomiasis vaccine is based on (a) the morbidity, mortality, and gender-based and socioeconomic implications of the illness, and (b) the global ambition to eliminate the disease (not just achieve its control).

Even with MDA, WASH, snail control, and other interventions, there is still a large burden of schistosomiasis. The Global Burden of Disease 2019 study found that schistosomiasis was responsible for 1.64 million DALYs. About 236.6 million people required treatment for schistosomiasis in 2019. Poor and rural communities are disproportionately affected. The risk is greatest in areas with poor access to safe water and adequate sanitation; high-risk activities include farming irrigated fields, fishing, washing cars, and washing clothes in infected water sources. Out-of-pocket costs associated with schistosomiasis have been poorly studied, but are likely to be considerable for severe disease. Cost of illness studies estimate that over 95% of total costs associated with schistosomiasis are due to productivity losses (e.g., lost wages due to illness and death). There are also productivity costs associated with livestock schistosomiasis. Schistosomiasis has multiple gendered dimensions—for example, FGS is a stigmatizing condition that affects reproductive health.

WHO launched a NTD Road Map for 2021–2030 that targets the elimination of schistosomiasis as a public health problem in all endemic countries. Under “actions required,” the Road Map states: “consider development of a vaccine for humans and animals to prevent reinfection and reduce transmission.” In addition to political and policy support for vaccine development, other supportive policy actors include the US NIH, the US Department of Defense, the Bill & Melinda Gates Foundation, the European Community, FIOCRUZ and the Brazilian Government, Australia’s NHMRC, SCI, and IVI.

Preferred product characteristics for a schistosomiasis vaccine, agnostic to a specific candidate, include: prevention of infection by one of the three human *Schistosoma* parasites (*S. mansoni*, *S. haematobium*, or *S. japonicum*); targets populations in endemic countries or regions, especially adults (18–59 years of age) in high-risk occupations or areas and high-risk school-age children (3–12 years of age); 2-dose, parenteral administration; at least 2–3 years of protection after last dosing; good safety profile; can be co-administered with praziquantel MDA and other interventions; storage at from − 20 ◦C to 4◦C; and stability expected to be over 3 years.

There are currently four vaccine candidates in clinical trials, described in detail earlier in this paper:

- **Bilhvax (Sh28GST vaccine)**, which has undergone phase 1–3 trials.
- The **Sm-14 Humanitarian Schistosomiasis Vaccine Initiative**, which has undergone Phase 1a and 1b in a non-endemic area of Brazil between 2010 and 2015; 2a and 2b trials in a Senegalese area endemic for both *S. mansoni* and *S. haematobium* conducted by EPLS between 2015 and 2019; followed by a Phase 2c trial which began in 2020 and is expected to reach conclusion in an endemic area in Senegal by the end of 2023. Safety was already attested.
- The **Human Schistosomiasis Vaccine**, which has undergone Phase 1 trials; a Phase 1/2 proof-of-efficacy trial in healthy exposed adults is currently underway in Uganda.
- **SchistoShield**^**®**^: A Phase 1a clinical trial has been completed in infection naïve adults in the US. No significant safety concerns have been observed in all of the groups including with dose escalation. Vaccine appears to be well tolerated and elicits pronounced vaccine-mediated responses. Phase 1b/2A dose-escalation trial among African adults, with a planned future age de-escalation study in school-aged children (Burkina Faso, Madagascar) is currently underway.

Several modelling studies have explored the use of a schistosomiasis vaccine and quantified its potential impact on disease burden and/or transmission. Key findings are: (i) vaccination used in combination with MDA would tend to provide greater benefits (e.g., the combination would reduce transmission more quickly) than using either alone; (ii) interrupting transmission should be more readily achieved in low transmission settings compared to moderate and high transmission settings; (iii) the duration of protection seems to be the largest driver of vaccination’s impact; (iv) mass vaccination of the community would more quickly interrupt transmission and has the greatest chances of achieving the WHO goals compared to vaccinating other target populations; and (v) vaccinating school-aged children seems to be the next best strategy, followed by infant vaccination (it would take several years to interrupt transmission in a community when only vaccinating infants as there is not a reduction in the current burden in the population when using this strategy).

Modelling studies have also estimated the socioeconomic impacts of a schistosomiasis vaccine. These have shown that a vaccine of at least 90% efficacy would be cost-effective and, in some circumstances, could generate cost savings, compared to MDA from the healthcare provider perspective. Vaccination would be more cost-effective than MDA, when the vaccine cost up to $9.20. However, this threshold cost varied by study and transmission setting (e.g., higher costing vaccines remained cost-effective in higher transmission settings) and ranged from $3 to $9.20. The duration of protection offered by a vaccine is a large driver of its cost-effectiveness and is a driver of which target population it is most cost-effective to vaccinate (e.g., with a shorter duration of protection it is more cost-effective to vaccinate school-aged children). Vaccination would be more cost-effective in higher transmission settings compared to lower transmission settings.

Finally, there is high overall feasibility when it comes to implementing schistosomiasis vaccines. First, they could be implemented within existing delivery systems and vaccines in current development have relatively simple dosing schedules. They could be co-administered with routine measles or preschool vaccines, alongside MDA programs, and with HPV vaccines. Schistosomiasis vaccines have moderate to high commercial attractiveness, since there is significant potential LMIC vaccine demand, many endemic countries are eligible for Gavi support (interest from Gavi has not been confirmed, but schistosomiasis vaccines are expected to fall within their remit), and there is also some HIC utility for military populations. Uptake is expected to be high, given the existing level of acceptance of vaccination, mass drug administration, and other health care approaches. Nevertheless, we note that Gavi’s procurement decisions are based primarily on deaths avoided, and the comparatively low mortality may not make this an attractive investment. The societal costs of morbidity need to be better enumerated to strengthen the case for donor funding of schistosomiasis vaccines (including in comparison to funding treatment). As we noted in the introduction, schistosomiasis vaccines also fit well with the One Health concept synergizing human and animal health: there are opportunities for using veterinary vaccines in the control of human schistosomiasis.

## Figures and Tables

**Table 1:** Summary of epidemiology and potential indirect public health impact.

Feature	Summary and evidence
***Epidemiology***	
**Reservoir**	*S. japonicum* and *S. mekongi* are established zoonotic species, while the African schistosome species, particularly *S. mansoni* and *S. haematobium* hybrids, are also now acknowledged to have a zoonotic component [6]. The key reservoir hosts for *S. japonicum* are, depending on country and habitat, water buffalo and cattle as well as rodents or dogs (but over 40 different mammalian species can serve as reservoirs)[7]. Dogs and domestic pigs are believed to be the key reservoir hosts for *S. mekongi* (other animals, including bovines, may also be involved in transmission). For *S. mansoni*, both rodents and non-human primates may serve as reservoir hosts [8], whilst for *S. haematobium* viable hybridization with livestock *Schistosoma* spp. also plays an important epidemiological role [9].
**At-risk populations**	Poor and rural communities are the most affected [2]. The general population who lives in affected areas and is exposed to contaminated water is at risk [3]. Endemic areas are characterized by insufficient sanitation and the presence of the intermediate host[4]. Certain activities put the population living in affected areas at increasing risk: those dependent in agriculture and fishing and domestic chores, like washing clothes, in infected water sources [2]. Adolescents and young adults (15–25 years) tend to have the highest infection intensities and can develop severe adverse effects (e.g., anaemia, pain) [10]. Severe portal hypertension and cancer can cause death, largely amongst the older age groups. The number of travellers to affected regions that come into contact with infected water and contract schistosomiasis is increasing [2].
**Mortality**	There is a wide range of estimates of the annual number of deaths worldwide due to schistosomiasis. In 2000, the WHO estimated there were 200,000 deaths globally, [11] whilst in 2016 the organization estimated that the number had fallen to 24,072.[12]
**Morbidity**	About 236.6 million people required treatment for schistosomiasis in 2019—mostly people living in poor, rural communities, especially fishing and agricultural communities [5]. The two main clinical types of African schistosomiasis are (i) intestinal/hepatic (caused by *S. mansoni*), causing symptoms such as diarrhea, abdominal pain, and blood in the stool, and (ii) urogenital (caused by *S. haematobium* and hybrids therein), causing symptoms such as blood in the urine, genital lesions and infertility in young women. *S. haematobium* is a urinary bladder carcinogen, and it may also play a role in other types of cancer, such as cervical cancer [13]. *S. intercalatum, S. guineensis*, mixed, and hybrid species infections do not give such a clear-cut morbidity profile [14,15]. Asian schistosomiasis is caused by three species: the most prevalent is *S. japonicum*, followed by *S. mekongi* and *S. malayensis* [16]. These may cause an acute illness (Katayama fever) weeks after the initial infection *(S. mansoni* can also cause Katayama fever, as can *S. haematobium*, but more rarely), with symptoms including fever, cough, and abdominal pain. Long term complications include hepatic and central nervous system disease (*S. japonicum* eggs in the brain can cause cerebral granulomatous disease). *Biomphalaria stramnia*, an intermediate host of *S. mansoni*, has been found in Guangdong province, southern China, and China has seen imported case of *S. mansoni* [17]. Female genital schistosomiasis (FGS), an inflammatory parasitic disease that is highly stigmatizing, is estimated to affect around 50 million people in *S. haematobium*-endemic areas of sub-Saharan Africa [18]. Genital schistosomiasis can also affect men, but the prevalence is unknown [13]. FGS causes inflammatory lesions, such as in the vulva, vagina, cervix, uterus, and ovaries, and can impair reproductive health. Symptoms include “contact and postcoital bleeding, genital itch, abnormal discharge, stress incontinence, and dyspareunia, leading to infertility in the longer term” [19]. Women with FGS have an elevated risk of HIV infection [20]: about a 3- to 4-fold risk compared with schistosomiasis-free women [19]. And HPV and *S. haematobium* may be “potential synergistic agents in cervical cancer pathophysiology” [13]. Intestinal schistosomiasis from *S. mansoni* can cause rectal lesions in women and men. The Global Burden of Disease 2019 study found that schistosomiasis was responsible for 1.64 million disability-adjusted life years (DALYs) [21]. However, there is considerable variation in morbidity estimates, likely due to methodological choices to include or exclude associated issues, such as undernutrition or anaemia [3]. Thanks in part to improved diagnostics and surveillance, we now know that among pre-school aged children, more of this very young age group are infected than previously assumed and the morbidity profiles may be different [22,23]. One 2013 study, for example, showed that over an 18-month period, in villages along the shore of Lake Victoria, Uganda, nearly half of the children aged 3 months and above had active infections [24].
**Geographical and seasonal distribution**	Schistosomiasis is prevalent in the tropics and subtropics and is endemic in 78 countries [2,3]. Most people (90%) who require treatment live in sub-Saharan Africa [2]. Schistosomiasis is a focal disease with varying prevalence even at a very local level (i. e., village to village), depending on factors such as community water contact patterns, the number of water sources available to a community, as well as host immunological and/or parasite genetic traits [3,25,26].
**Gender distribution**	While studies report differences in prevalence of infection between men and women, a recent systematic review found that many of these findings are not statistically significant [27]. Prevalence of infection potentially reflects gender norms and the water contact activities associated with them (e.g., fishing, clothes washing, agriculture). Specifically, the systematic review identified four major risk factors for infection: occupational and recreational water contact, knowledge of the disease and its transmission, socio-economic factors (e.g., income, occupation of parents) and demographic factors (e.g., age) [27]. Although praziquantel is safe for pregnant and lactating women, this population has often been excluded from MDA campaigns despite the fact that women in endemic areas spend “up to 25% of their reproductive years pregnant and another 60% of this time lactating [10,28].” Chronic schistosomiasis infection that is not consistently treated due to pregnancy and breastfeeding may increase susceptibility to organ damage and cancer [29]. According to the WHO, “services are infrequently adapted to meet the needs of pregnant women and therefore they may be left untreated,” which puts them at greater risk of disease-associated morbidities [30]. Given that FGS is a stigmatizing condition that affects reproductive health, Hotez and colleagues argue that a package of interventions is needed to tackle three overlapping health challenges [19]. The first is lack of access to praziquantel. The second is the social stigma associated with FGS. The third is “the failure to consider FGS as a central element of sexual and reproductive health,” an important social justice issue.
**Socio-economic status vulnerability(ies) (equity/wealth quintile)**	Schistosomiasis is considered a poverty-related disease, and most individuals with active and late chronic disease are from poor rural areas, particularly agricultural and fishing populations [2,3]. A study in Uganda on the association between schistosomiasis and socioeconomic status, which controlled for access to treatment and water contact, found that wealth remains an independent predictor of infection and intensity [31].
**Natural immunity**	In endemic areas where exposure to infection is constant, worm burdens, and thereby the acute severity of symptoms, tend to decline from teenage years when some immunity develops [3]. Protective immunity develops slowly—typically over a period of 10–15 years. In endemic areas, adults are usually resistant to reinfection, whereas children under 10 can get re-infected after treatment. Immune responses towards cercariae (the “free-swimming larval stage in which the parasites pass from the intermediate to the final host” [3]) are thought to be responsible for resistance to reinfection; in contrast, schistosomiasis pathology is largely due to responses towards egg antigens. The issue of immunology or behaviour still is uncertain in determining if the convex age infection intensity curves are due to acquired immunity or behaviour that influences exposure to infection. Both are likely to play some role but strong acquired immunity is absent as post praziquantel treatment adults can still acquire new infections. One hypothesis to explain the development of natural immunity is that it relates to the death of adult worms, either naturally or after praziquantel treatment. The hypothesis is that such death causes release of antigens that “may cross-react with larval antigens and stimulate protective IgE responses” [3]—these responses are associated with resistance to reinfection [32]. Experiencing more dead worms would therefore elicit stronger protective responses.
**Pathogenic types, strains, and serotypes**	There are six, largely geographically distinct, species of *Schistosoma* that infect humans, three of which are the main cause of disease in humans (*S. haematobium*, and hybrids therein, *S. mansoni*, and *S. japonicum*). The other three (*S. guineensis, S. intercalatum* and *S. mekongi*) are more geographically localized [1,3]. • The most common species is *S. haematobium* (and/or hybrids therein [33]), reported in 54 countries, mostly in sub-Saharan Africa and the Middle East; there are also sporadic outbreaks in Europe, most likely to be due to *S. haematobium* hybrids • *S. mansoni* is endemic in sub-Saharan Africa, Brazil, the Caribbean islands, Puerto Rico, Suriname and Venezuela • *S. japonicum* is endemic in China and the Philippines, with small foci in Indonesia (Japan eliminated it in the late 1970s) • *S. guineensis* and *S. intercalatum* are endemic in West Africa and Central Africa • *S. mekongi* is restricted to the southern parts of Cambodia and along the Mekong river in Lao People’s Democratic Republic.
***Potential indirect impact***	
**Anti-microbial resistance (AMR) threat**	Praziquantel is the only effective antischistosomal drug currently used to treat infection. Its frequency of use, as well as use among broad population groups (now including children and pregnant women), raises concerns about resistance developing in the absence of another viable treatment available [34,35].
**Epidemic and outbreak potential**	The WHO notes that schistosomiasis is endemic in 78 countries. Mass chemotherapy is used in 51 endemic countries with moderate-to-high transmission [2]. Notable schistosomiasis outbreaks that have been reported, include in, for example, Corsica, Malawi, and Senegal [36–38]: • In 2014, cases of urogenital schistosomiasis in German and French tourists were traced back to a river in Corsica, an island off the coast of southern France. The outbreak may have been due to a hybrid schistosome—a combination of *S. haematobium* with *S. bovis* (a livestock parasite). The schistosomes were probably imported from west Africa (specifically Senegal). • In 2020, school children in the Mangochi district of Lake Malawi experienced an outbreak of intestinal schistosomiasis. The lake is endemic for urogenital schistosomiasis (the *Bulinus* snail host lives along its shoreline) but not intestinal schistosomiasis. *S. mansoni* was confirmed, along with “the unexpected discovery of another snail host species, *Biomphalaria pfeifferi* [39].” O’Ferrall and colleagues note that “the threat of increased transmission and morbidity associated with both forms of schistosomiasis in Mangochi District now exists.” • Intestinal schistosomiasis had never been detected in the Senegal river basin until an outbreak in 1988–1989 (urogenital schistosomiasis had been endemic). This outbreak was related to the building of the Diama dam, which blocked intrusion of saltwater into the river and created more suitable breeding sites for the intermediate host snails, combined with migration into the area. There are also *S. mansoni* morbidity hotspots, with outbreaks, around the Lake Albert region of Uganda, the reasons for which are as yet to be fully determined. Boissier and colleagues note that “compatible snail vectors are endemic in several European countries,” which means there could be further outbreaks through the importation of schistosomes [36].
**Transmission route/potential**	Humans become infected when exposed to contaminated fresh water. Contaminated water means that a snail (i.e., the intermediate host) has released cercariae (i.e., the infectious stage of schistosomes) into the water source [3]. Cercariae then infect their human hosts by penetrating their skin [3]. The cycle is repeated when an infected human excretes into the water source (urine or feces), releasing free-swimming larvae that infect host snails [3]. Given the importance of environmental context for the spread of schistosomiasis, climate change and other anthropogenic changes may affect transmission in the future through affecting aquatic environments [3].
**Acquired/herd immunity**	Partial immunity to schistosomiasis may happen after a long period of time for those living in endemic regions [40]. Age is a major factor in immunity: children under the age of eleven are more susceptible to infection/reinfection than adults [40]. Immunity occurs through the death of adult worms, which can either happen naturally or with treatment using praziquantel [40]. However, various individuals factors can affect the study of immunity at a given time (e.g., currently infected/uninfected, treatment with praziquantel, if their mother was infected while they were in utero, etc.) [40]. Immunity is still not fully understood and requires more study. Behavioural studies show a strong correlation with water exposure and the intensity of infection by age group, so the convex patterns of the intensity of infection by age will be a combination of exposure and the slow build-up of past experience of infection.
**Co-associated mortality/morbidity**	Schistosome eggs that are not excreted and that are trapped in human tissue can cause chronic inflammation, which leads to health problems such as anemia, growth deficiencies, diarrhea, undernutrition, fatigue, exercise intolerance, and impaired cognitive development [41,42]. Female genital schistosomiasis is also associated with an increased risk of HIV infection and other sexually-transmitted infections [42].
***Economic burden***	
**Health facility costs/out of pocket costs/productivity costs**	A 2020 systematic review found considerable variation in the per person treatment costs, from US$0.06–$4.46 [43]. These per person treatment costs were further broken down as follows: • Chemotherapy interventions: $0.05–4.46 • Chemotherapy plus an individual diagnostic test to identify at-risk populations: $1.19–4.45 • Test-and-treat interventions: $0.35–$2.51. Out-of-pocket (OOP) costs have been poorly studied, but are likely to be considerable for severe disease. For example, in a study in Hunan Province, China, researchers interviewed 79 patients with advanced schistosomiasis japonica—none had health insurance and they faced high OOP expenses [44]. A 2016 systematic review found 10 studies that estimated the productivity costs of human schistosomiasis—the estimates varied widely based on factors such as the severity and duration of illness, the disease sequelae, and the type of schistosomiasis [45]. Studies generally compared productivity losses between infected and uninfected workers, but used different units of losses e.g., lost work days, lost work hours, reduced earnings. The authors of the systematic review translated the study findings into a percentage of annual productivity loss assuming 300 working days a year. The percentage loss ranged from 1% (in two studies, one of *S. haematobium* in Niger and one of *S. japonicum* in China) to 100% for very severe schistosomiasis (e.g., in a study of *S. japonicum* in the Philippines). In addition, livestock schistosomiasis also affects productivity. For example, Adeyemo and colleagues estimated the financial impact of livestock schistosomiasis in Senegal. They found that “the median disease costs in a representative herd for the areas studied were between 0.23 and 1.22 of the average annual income in rural Senegal.”[46]

**Table 2:** Overview of potential target and key population(s) and associated delivery strategy(ies).

Target and key population(s)	Delivery strategy(ies)

Pre-school aged children (PSAC, 1–5 year old)	As part of the routine childhood Expanded Programme on Immunization (EPI) in health centres (primary use case) [57]
School aged children (SAC) and adolescents (5–18 year old)	If in school: routine school-based delivery programmes (primary use case) If out of school: targeted campaigns for out-of-school children and adolescents (primary use case) [57]
Adults (general population, 18 and above)	Adult vaccination offered when adults bring children to health centres for EPI (complementary use to expand access and coverage) [57]
Adults (high risk groups such as paddy-field workers, farmers OR from high-endemicity areas)	Targeted vaccination campaigns for high-risk groups and high endemicity areas (complementary use to expand access and coverage)
Women of child-bearing age	Vaccination offered as part of maternal and neonatal visits in health centres (complementary use to expand access and coverage)

**Table 3:** Overview of non-commercial stakeholders engaged, their interest and potential demand.

Stakeholders engaged	Summary of position/interest	Potential demand and uptake

World Health Organization [6]	• WHO has developed a strategy on use of anthelminthic drugs, which currently focuses on control of schistosomiasis in conjunction with interventions against lymphatic filariasis, onchocerciasis and soil-transmitted helminthiasis. • WHO is also collaborating with partners to support knowledge translation on schistosomiasis vaccine development.	• According to WHO estimates, at least 236.6 million people required preventive treatment for schistosomiasis in 2019, out of which more than 105.4 million people were reported to have been treated. This represents significant population numbers, suggesting there could be considerable demand for a vaccine in LMICs [62].
Oswaldo Cruz Foundation (FIOCRUZ) and Brazilian Health Ministry, a South- South innovative venue towards the development of anti-helminth vaccines and mitigation of health inequities between HICs and LMICs	• Supports the Schistosomiasis Vaccine Initiative and the development of the Sm14-based anti-helminth vaccine since the 1980s. Since 2021, FIOCRUZ decided to become the sole supporter of the final development of the Sm14 schistosomiasis vaccine with public funds and to coordinate the vaccine initiative under a humanitarian vaccine policy.	• Funds from the Brazilian government and Brazilian financial agencies (e.g., Finep) in association with private entities (e.g., Orygen Biotechnology) financed the phase 2a clinical trial in adults in the Senegal river delta region. Supports the Sm14 vaccine initiative up to the present. The public-private entities are strongly committed to this the South-South innovative program. • Since 2021, Biomanguinhos/Fiocruz took over the production of the Sm14 human vaccine against schistosomiasis under a humanitarian vaccine profile and production policy.
US National Institutes of Health [63]	• Operates the Schistosomiasis Research Center, which focuses on contributing to the development of schistosomiasis research (NIAID). • Infectious Diseases Clinical Research Consortium, a clinical trials network, has funded/funds phase 1 trials of schistosomiasis vaccine (NIAID). • Tropical Medicine Research Centers (TMRCs) in disease-endemic countries (NIAID).	• Uptake/demand is primarily located in low- and middle-income countries; there is some U.S. interest for travellers and military populations. The TMRCs are designed to conduct research on the cause, diagnosis, prevention, and treatment of NTDs, and to build in-country research capacity.
US Department of Defence	• US military conducts activities in helminth-endemic regions, including Africa, the Middle East and Southeast Asia. However, it does not currently screen military members and families for infections or conduct routine surveillance in this population.	• Burden of helminth infections in U.S. military personnel and dependents is unknown, but infections have been diagnosed in military populations. [64,65]
Bill & Melinda Gates Foundation [66]	• Works with partners to develop tools for neglected tropical diseases, including schistosomiasis. • Areas of focus include public health surveillance, vector control, and MDA. • Significant funder of the SchistoShield^®^ anti-schistosomiasis vaccine in adults in endemic areas of sub-Saharan Africa (Phase 2a).	• See above for demand in LMICs.
European Commission	• Significant funder of the SchistoShield^®^ anti-schistosomiasis vaccine in adults in endemic areas of sub-Saharan Africa (see Table 6) [67].	• See above for demand in LMICs.
Australian National Health and Medical Research Council (NHMRC)	• Significant funder of schistosomiasis vaccine research.	• See above for demand in LMICs.
Unlimit Health (previously the SCI or Schistosomiasis Control Initiative Foundation) [68]	• Focuses on public health programmes that work towards the elimination of preventable diseases, including schistosomiasis.	• See above for demand in LMICs.
International Vaccine Institute [69]	Schistosomiasis projects include: • Schistosomiasis in Madagascar (SOMA) project in collaboration with the University of Antananarivo, which aims to reduce the intensity and prevalence of schistosomiasis infection for at-risk populations in a target area. • **Vaccine Against Schistosomiasis for Africa** (VASA) project in collaboration with Cambridge University. The project focuses on understanding the current disease burden in Madagascar and Burkina Faso; understanding the financial burden of schistosomiasis on local populations with a cost-of-illness study; estimating the cost-effectiveness of a vaccine; and conducting a Phase I clinical trial to assess the safety and immunogenicity of SchistoShield^®^. • Phase 2a clinical study of Sm-p80 vaccine for schistosomiasis.	• See above for demand in LMICs.
RIGHT Foundation	• Significant funder of schistosomiasis vaccine manufacturing and GMP production.	• See above for demand in LMICs.
Wellcome Trust	• Development of schistosome human challenge model for vaccine testing.	• See above for demand in LMICs.

**Table 4:** Summary of existing guidance on preferences for product attributes of vaccines intended for use in LMICs.

Product attribute	Minimal characteristic, if described	Preferential characteristic	Publishing entity

Indication	Prevention of infection by one of the three human *Schistosoma* parasites (i.e., *S. mansoni, S. haematobium*, or *S. japonicum*).	Prevention of infection and treatment of *Schistosomiasis* caused by all *S. mansoni, S. haematobium* (and hybrids therein), and *S. japonicum* parasites.	Division of Microbiology and Infectious Diseases, National Institute of Allergy and Infectious Diseases, National Institute of Health, Bethesda, MD, USA [57,70].
Target population(s)	Populations in endemic countries or regions, especially adults (18–59 years of age) in high-risk occupations or areas and high-risk school-age children (3–12 years of age).	Populations in endemic countries or regions ≥1 years of age.	[57,71]
Outcome measure(s) and target efficacy	Prevent at least 60% RE – infection by one of the human *Schistosoma* species. Outcome measure: egg output and/or worm burden.	Prevent at least 60–70% RE-infection caused by all human *Schistosoma* species. Outcome measure: egg output and/or worm burden.	[57]
Safety profile	Local mild site reactions; incidence of serious adverse events (SAEs) or increased risk of autoimmune or chronic inflammatory diseases comparable to other licenced vaccines.	Mild local injection site reactions such as erythema, oedema and pain, the character, frequency, and severity of which is similar to licensed vaccines. Less than 0.001% risk of urticaria and other systemic allergic reactions. Incidence of SAEs and autoimmune or chronic inflammatory diseases is no more than licensed comparator vaccines.	[57]
Number of doses and schedule	2–3 dose administration. The need for booster doses in endemic areas will be determined after the 12-months follow up after the vaccination campaign.	3 dose administration, included in the children’s calendar, following hepatitis B vaccination schedule.	[57]
Route of administration	Parenteral administration (e.g., intramuscular).	Intramuscular.	[57]
Duration of protection	2–3 years after last dosing.	5–10 years after last dose.	[57]
Co-administration with other vaccine	Can be co-administered with praziquantel MDA and other interventions. No interference or adverse interactions with: • Prior or subsequent local anti-helminth drug treatment (e.g., PZQ) • Co-administered with any EPI vaccines • Prior or subsequent routine child and adult vaccines within a 2-week window.	Same.	[57,72]
Product stability and storage	Storage at from −20°C to 4°C. Stability expected to be >3 years and more.	Storage at 2–8°C or room temperature. Stable for ≥10 years.	[57,72]
Vaccine presentation	Vaccine is provided as a liquid product in single-dose presentations with a maximal dosage volume of 2.0 mL. Vaccine should be formulated, managed, and discarded in compliance with biomedical waste disposal standards.	Vaccine is provided as a liquid or lyophilized product in single- or multi-dose (e.g., 10–20 dose) vials with a maximal dosage volume of 1.0 mL. Lyophilized vaccine will need to be accompanied by paired separate vials of the appropriate diluent. Vaccine should be formulated, managed and discarded in compliance with biomedical waste disposal standards.	[57,72]

**Table 5:** Overview of parameters that inform scientific feasibility of developing an effective vaccine for LMIC public market use.

Parameter	Issues and evidence

Diagnosis/case ascertainment	Cases of intestinal/hepatic schistosomiasis (*S. mansoni*) are principally determined by microscopic examination of fresh fecal samples. Cases of urinary schistosomiasis (*S. haematobium*) are principally determined by microscopic examination of fresh urine samples [92]. Additional methods of detection include measurement of Circulating Anodic Antigen (CAA) in serum or urine samples, Circulating Cathodic Antigen (CCA) in serum or urine samples (point-of-care testing for the latter), or PCR of stool or urine (both *S. mansoni* and *S. haematobium*) [93].
Biomarkers/correlates of risk and/or protection	There is no established correlate of protection or risk. In all likelihood several serum-based and molecular readouts as the surrogate(s) of composite efficacy will need to be identified. In rodent and nonhuman primate models, IgG levels at challenge tend to negatively correlate with parasite burden in all of the schistosomiasis vaccines in clinical trials.
Sero-epidemiological data	Recombinant *Sm*-TSP-2, but not *Sm*-TSP-1, is strongly recognized by IgG1 and IgG3 (but not IgE) from naturally resistant (“putatively resistant”) individuals but is not recognized by IgG from chronically infected or unexposed individuals [94].
Clinical endpoints	Potential purely clinical endpoints for *S. mansoni* vaccines include anaemia, hepatic fibrosis, portal hypertension, oesophageal varices and splenomegaly. However, these endpoints usually occur late in disease progression, often only after a decade or longer of chronic infection. Alternative proposed efficacy endpoints for pivotal Phase 3 trials include moderate or heavy intensity infection defined by semiquantitative fecal egg counts (eggs per gram [EPG] of feces) [95]. Potential clinical endpoints for *S. haematobium* vaccines include: (micro)haematuria, anaemia. The single Phase 3 trial completed to date of a vaccine targeting *S. haematobium* (rSh28GST) used a primary efficacy endpoint of microhaematuria in the presence of at least one egg on microscopic urine examination.
Controlled human infection model (CHIM)	A controlled human infection model (CHIM) for *S. mansoni* has been developed at Leiden University Medical Center (see below for a discussion of CHIM). This has used single-sex cercariae to induce infection in healthy, schistosomiasis-naïve adults. A CHIM could be used to down-select potential vaccine antigens but it has not yet been used for this purpose. There are concerns about the relevance of single-sex infection to natural infection [96].
Opportunity for innovative clinical trial designs	Among the potential innovations in trial designs for efficacy would be evaluating a multivalent vaccine targeting more than one Schistosoma species (e.g., *S. mansoni* and *S. haematobium*) in areas of co-endemicity and high transmission. In this setting of high attack rates, it is potentially an option to get a rapid answer on vaccine efficacy using reductions in egg intensity (quantitative fecal or urine egg counts) relative to controls.
Regulatory approach(es), including potential accelerated approval strategies	Stringent national regulatory authority (NRA) review followed by WHO pre-qualification (PQ). Initial target countries for early registration are Brazil, Uganda, Nigeria, Ethiopia, and the Democratic Republic of Congo.
Potential for combination with other vaccines	The idea of combining antigens includes untested variables, parameters that could demand long-term development. That could be planned as a second generation of anti-schistosomiasis vaccines in well-known and safe production platforms.
Feasibility of meeting presentation and stability requirements	A key issue is whether the vaccine can achieve the presentation, storage and stability requirements, considering the intended delivery setting and strategy
Vaccine platform	Adjuvanted recombinant proteins are easy to scale up for large volume manufacture.
Large scale manufacturer capacity/interest	• Instituto Butantan in Brazil has previously signed a memorandum of understanding (MOU) to scale and commercialize the *Sm*-TSP-2 vaccine for the Americas. • Oswaldo Cruz Foundation in Brazil is committed to scale and commercialize the *Sm14* vaccine globally with the profile of a humanitarian vaccine.

**Table 6:** Overview of vaccine candidates in clinical trials.

Candidate	Antigen platform	Developer/manufacturer	Phase of development, population, and location	Route of administration, no. of doses, schedule	Clinical trial references

The Human Schistosomiasis Vaccine	*Sm*-TSP-2 (Tetraspanin in schistosome tegument); recombinant protein produced in yeast	Texas Children’s Hospital Center for Vaccine Development; IBT Biologika (former AERAS), Vaccine and Treatment Evaluation Unit (VTEU), Baylor College of Medicine; George Washington University; FIOCRUZ; James Cook University; Access to Advanced Health Institute (AAHI) [former Infectious Disease Research Institute (IDRI)]; Makerere University Walter Reed Project (MUWRP)	Phase 1 trials conducted in 2016 in both endemic and non-endemic settings, amongst adults. In both cases, the vaccine was safe and well-tolerated with no vaccine-related adverse events. Antigen-specific IgG antibodies were induced in a dose-dependent fashion. A DOD-CDMRP supported Phase 1/2 proof-of-efficacy trial in healthy exposed adults is currently underway in Uganda.	The Phase 1 trial in a non-endemic setting tested two formulations of *Sm*-TSP-2 vaccine (using Alhydrogel only as an adjuvant, and using Alhydrogel plus a second adjuvant, GLA-AF [glucopyranosyl lipid adjuvant in an aqueous formulation]), each at 3 different doses: 10ug, 30ug, and 100ug. The route of administration was i.m., administered in three doses on Days 1, 57, and 113. The schedule is the same for the Phase 1 trial in Brazil and Phase 1/2 trial in Uganda, with the second also GLA-AF (re-named “AP 10–701”).	[97–99] https://clinicaltrials.gov/ct2/show/NCT02337855 (phase 1, non-endemic setting) https://clinicaltrials.gov/ct2/show/NCT03110757 (phase 1 trial, endemic setting in Brazil) https://clinicaltrials.gov/ct2/show/NCT03910972 (phase 1/2 trial in Uganda)
SchistoShield ^®^	Sm-p80 (Calpain in schistosome apical membrane)	Texas Tech University Health Sciences Center; AAHI (IDRI); PAI Life Sciences	A phase 1a clinical trial using Sm-p80 formulated in GLA-SE (glucopyranosyl lipid A stable emulsion) has been completed in infection naïve adults in the US. The Phase 1b dose-escalation trial among African adults, with a planned future age de-escalation study in school-aged children, is currently underway in Madagascar and Burkina Faso.	The route of administration is i.m. The Sm-p80 vaccine is being tested alone or with the GLA-SE adjuvant. The trial has assessed safety and reactogenicity following receipt of three doses of 1) 100 micrograms Sm-p80 (unadjuvanted), 2) 10 micrograms Sm-p80 + 5 micrograms GLA-SE, 3) 30 micrograms Sm-p80 + 5 micrograms GLA- SE, and 4) 100 micrograms Sm-p80 + 5 micrograms GLA-SE administered intramuscularly on Days 1, 29, and 57 and 5) 30 micrograms Sm-p80 + 5 micrograms GLA- SE administered on Days 1, 29, and 180.	[100] https://clinicaltrials.gov/ct2/show/NCT05292391 (phase 1 trial)
The Sm-14 Humanitarian Schistosomiasis Vaccine Initiative	Sm-14 (fatty acid binding protein)	FIOCRUZ, Brazilian Health Ministry, Biomanguinhos	A Phase 1 trial in healthy subjects from a nonendemic area of Brazil found that the vaccine was highly immunogenic. It was well-tolerated and there were no detectable vaccine-induced IgE antibodies. [1] A follow-up phase 2a study among adults in a schistosome-endemic region of Senegal showed that Sm14/GLASE was safe and resulted in 92% seroconversion after the third immunization. [1] Based on these results, a phase 2b study in school-aged children living in the same endemic area of Senegal was conducted and completed in 2019. The results are yet to be released.	The route of administration is i.m. Phase 1 was developed in a nonendemic area of Rio de Janeiro in two independent trials: first in men and then in women. Volunteers received three doses of Sm14 vaccine, in doses containing 50 mcg of the antigen, associated with adjuvant GLA-SE at a dose of 10 mcg, with an interval of 30 days between each application. Both Phase 1 trials were licensed by ANVISA (Brazilian Ministry of Health), financed by Finep, and conducted by the National Institute of Infectious Diseases (INI)/FIOCRUZ between 2010 and 2015. Phase 2 trials were conducted by Espoir Pour La Santé (EPLS), a Franco-Senegalese organization, at the Island of Saint Louis, at the Senegal river delta basin. In the phase 2a trial in adults, there were two parallel arms; each received three injections at day 0, day 28, and day 56; both groups received 50 μg Sm14 vaccine candidate solution, either combined with 2.5 μg GLA-SE for the first group and 5 μg for the second one in adults living in a *S. mansoni* and *S. haematobium* endemic areas. In addition, a Phase 2a extension trial was performed with the same groups but under a specific license to confirm the duration of immunity to up to 12 months after the first vaccination. In the phase 2b trial, performed by EPLS in school children of different villages of the same region of the Phase 2a, there were three parallel arms, two of them formed by groups of healthy or infected school children, both receiving three injections at day 0, week 4, and week 8; both groups received 50 μg Sm14 vaccine candidate solution, combined with 2.5 μg GLA-SE. The third group was composed of non-vaccinated infected school children. Phase 2a and 2b were conducted between 2015 and 2019. Phase 2c began in Senegal in 2020 and will conclude in 2023. It presents an alternative vaccination schedule known to be more immunogenic. The GMP Sm14 lot used was produced at a facility of the University of Nebraska-Lincoln.	[101–103] https://clinicaltrials.gov/ct2/show/NCT01154049 (Phase 1 trial) https://clinicaltrials.gov/ct2/show/NCT03041766 (Phase 2a trial in Senegal) https://clinicaltrials.gov/ct2/show/study/NCT03799510 (Phase 2b trial in Senegal)
Bilhvax (Sh28GST Vaccine)	Sh28GST (recombinant 28 kDa glutathione S-transferase of *Schistosoma haematobium*)	Phase 1: University Hospital, Lille and Institut National de la Santé Et de la Recherche Médicale, France; Phase 2 and 3 trials: Institut National de la Santé Et de la Recherche Médicale, France, and ESPOIR Pour La Santé, Senegal	Phase 1 study of recombinant Sh28GST adsorbed to Alhydrogel (Bilhvax) in healthy adults completed; vaccine was well-tolerated and elicited a strong Th2-biased immune response. Phase 2 trial of Bilhvax and praziquantel (PZQ) in *S. haematobium*-infected adults and children found that the vaccine was safe. Phase 3 trial evaluated Bilharvax in PZQ-treated infected school-aged children in Senegal, but found suboptimal efficacy levels.	The route of administration is subcutaneous. In one arm of the trial, volunteers received 3 administrations of 100 μg of rSh28GST together with aluminium hydroxide (Alum) as adjuvant at day 0, day 28 and day 150. In the second arm, they received 2 administrations of 300 μg of rSh28GST together with Alum as adjuvant at day 0 and day 28.	[95,104,105] https://www.clinicaltrials.gov/ct2/show/NCT01512277 (phase 1 trial)

**Table 7:** Overview of modelling studies that measure health impact on disease burden and transmission.

Policy question	Assessment method/measure	Additional information specific to models	Assumptions	Outcomes/interpretation

What are the potential long-term consequences of sustained large-scale vaccination? [110]	Vaccine effectiveness was assessed by estimating the impact on infection rates (mean egg count) at different ages	–Model represents dynamics of mean worm burden and egg output per host with host age and through time – The model structure and parameter values were chosen to represent the population biology of *S. mansoni* (qualitative results may apply to other schistosome species) –Rates of transmission are related to levels of infection in population by a density-dependent function –Exposure to infection is age-dependent with peak exposure at 15-years old	–Vaccine assumed to provide 75% protection against infection or 75% reduction in egg output per worm, or a combination of 50% protection against infection and a 50% reduction in egg output per worm (consistent with animal models) – Mean duration of vaccine protection was 10 years – Infection-acquired (natural) immunity develops gradually and provides partial protection that may decay in the absence of continued exposure –Vaccine-induced immunity reduces infection rates, or reduces egg output, or a combination of both – Chemotherapy instantaneously reduced per capital worm burden by 95% –80% coverage of target population for both vaccination and chemotherapy –Scenarios looked at targeting 1-year old children (i.e., inclusion of vaccination within the EPI program), and 7-year-olds (i.e., school-aged children)	–Vaccinating 1-year olds reduces the rate of infection by 75% and reduces infection levels in the unvaccinated – Vaccines acting to reduce infection rates or egg output will have a similar impact on levels of infection – Vaccine impact may be highly sensitive to the duration as well as the degree of protection – Vaccination shifts the peak levels of infection to older age groups – Delaying vaccination until age 7 resulted in greater reductions in overall levels of infection and in peak levels of infection than vaccination at 1 year old –Combining vaccination with MDA was more effective than either alone. When combined in the 1st year (covering 80% of all age classes, removing 95% of infection from those treated), then levels of infection over the first 20 years are substantially reduced –Vaccination alone will not eliminate schistosome infection
What would be the impact of a vaccine with an efficacy of 100% when applied in endemic regions with different intensities of transmission? [111]	–Probability of achieving morbidity control and elimination (more specifically, the probability of achieving the WHO goals) – Assessed by evaluating the fraction of school-aged children (SAC) with heavy intensity infection prevalence (≤5% heavy-intensity infection in SAC for the morbidity goal and ≤1% heavy-intensity infection in SAC for the elimination as a public health problem goal)	– Individual-based age-structured stochastic model allowing for the probability of a given event occurring –Individuals can be vaccinated or unvaccinated –Represents *S. mansoni* in Iiteune, Kenya	– The model considers “an ideal case-perfect vaccine” such that the rate of infection and the rate of egg production are essentially reduced by 100% – Vaccination is given annually to the children with the pre-specified age of administration, and the coverage levels depend on the age group that is treated and the duration of vaccine protection –Evaluated two durations of protection, 5 and 20 years –Scenarios varied the target population, vaccinating SAC (5–15 years old) or 1-year olds – For a vaccine that provides a 20-year protection, vaccination occurs at age 1 (early start) with an 85% coverage or age 5 (school start) with 1 60% coverage, based on coverages of HPV and DTP vaccines –For a vaccine that provides 5 years of protection against infection, scenarios assume continuous protection, vaccinating either at ages 1, 6, and 11 with coverage levels of 85%, 60%, and 70% respectively, or at ages 5, 10, and 15 with coverage levels of 60%, 70% and 45%, respectively –Scenarios considered MDA and vaccination, alone or in combination –Evaluated in 3 different transmission settings as defined by WHO on the basis of prevalence; low (<10% baseline prevalence among SAC), moderate (10–50% baseline prevalence among SAC) and high (≥50% baseline prevalence among SAC) settings	–The probability of achieving morbidity control and elimination as a public health problem depends on the duration of protection provided by vaccination, the age of those vaccinated, and the coverage levels achieved – In low prevalence settings, WHO goals can be achieved for all treatment strategies (>90% probability of achieving by year 15 with vaccination alone) –In moderate prevalence settings, a vaccine that provides 5 years of protection can achieve both goals within 15 years of treatment – In high prevalence settings, by vaccinating at age 1, 6 and 11, can achieve morbidity control with an 89% probability but cannot achieve elimination –A combined vaccination and MDA strategy has the greatest chance of achieving the WHO goals in the shorter term – Results suggest that in order to achieve elimination as a public health problem, combining vaccination (5 years of protection) with MDA (treating 75% of SAC) is the best option
What vaccination coverage is required to achieve elimination, assuming a partially effective vaccine? [112]	–Impact of vaccine on community level outcomes –Accounts for indirect effects on unvaccinated persons (i.e., herd immunity impact) –Assesses proportion of infants successfully vaccinated to achieve interruption of transmission (i.e., critical infant vaccination coverage)	–Deterministic mathematical model – The model focuses on infant vaccination, immunizing within the first year of life before acquiring infection – Vaccine acts on adult worm mortality, fecundity, and/or establishment –While, uncertainty of key parasite population parameters exists, analyses use consensus for parameter values –Age-dependent infection rates are fit to observed age intensity and age prevalence profiles for *S. mansoni*	–Partial vaccine efficacy, varied in analyses from 0%–100% –Explored range of effective reproductive numbers (1–10), representing different transmission levels in the community –Varied duration of protection from 1 to 50 years	– A vaccine with efficacy of over 60% can interrupt transmission in communities with low and moderate transmission – In higher transmission settings, greater vaccine efficacy or higher infant vaccination coverage or multiple booster vaccine doses each year may be necessary. For example, 100% efficacy with 75% coverage to break transmission (assuming life-long protection duration) – Breaking transmission, even in low intensity transmission areas, may take 18 years or more when vaccinating infants – The duration of protection is an important driver of results. If less than a few years (e.g., less than 5), repeated immunisation may be necessary –Results show an average duration of protection of 5–10 years, which is believed to be adequate for good community impact with appropriate coverage levels
Quantify the population-level impact of a vaccine administered alone or in combination with MDA and determine factors in vaccine design and implementation to optimize role of vaccine in control and elimination [113]	–Estimated possibility of elimination (WHO definition of reaching and maintaining zero incidence) –Incidence measured as number of new worms acquired per person per year –Prevalence over time	–Deterministic compartment model, stratified by vaccination status, age, and worm burden –Model represents human and snail populations –Populated and calibrated with data for endemic villages in coastal Kenya –Parameterized to represent *S. haematobium* transmission	–High-risk Kenyan community –3 types of vaccine efficacy (reducing work accumulation, i.e., susceptibility; increasing worm mortality; reducing number of eggs produced, i.e., fecundity), base case for each set at 80% (based on TPP) –Base case duration of action set at 10 years –Target populations: childhood vaccination (i.e., newborns) and mass vaccination of all unvaccinated people –Evaluated in different endemic settings for 30 years	–In a high-prevalence community, 3 rounds of mass vaccination (80% efficacies) reduced human prevalence by 56%, snail prevalence by 24%, mean intensity of infection by 87%, and incidence by 73% with universal coverage –Vaccinating only children would be difficult to effectively interrupt transmission at an early date –Number needed to vaccination per worm averted increased with decreases in endemic intensity –Population-level impact of vaccine increases with vaccination coverage, water contamination, and continuation of high coverage over time –Mass vaccination in high burden settings will be necessary
Investigate the cost-effectiveness of a potential vaccine and calculate a critical vaccination cost [114]	–Prevalence and heavy intensity infection prevalence among school-aged children and adults over 30 years	–Stochastic individual-based transmission model with *S. mansoni* explicitly transmitted between human hosts and external reservoir –Matched to data from epidemiological studies in Kenya	–Vaccine reduces establishment of new worms by 90% and reduces fecundity by 90% –Varied duration of protection (2.5–20 years) –Cohort delivery (annual vaccination of children in particular age groups) and larger group delivery (e.g., school-aged children administered based on duration of protection)	–Eradication of heavy intensity infection after 15 years occurs when vaccinating 75% of school-aged children and 40% of adults –Interrupted transmission not achieved within 30 years when vaccine provides protection for 10 years and is delivered to children at age 1 and 10 years
Is a vaccine alone or in combination with a drug treatment a more effective strategy to achieve the goals of the World Health Organization 2021–2030 NTD road map? [115]	– Effectiveness of control strategies measured as the probability of reaching WHO morbidity and elimination goals with 15 years – Prevalence of heavy intensity infections over 15 years	–Used two different models –Both individual-based transmission models	–Vaccine reduces rate of infection and rate of egg production by 100% – Varied duration of protection from 5–20 years –Vaccination strategies evaluated: cohort with catch-up campaign; entire age groups in population; cohort vaccination with MDA – Frequency of vaccination varied with duration of protection	– In a low transmission setting all strategies achieved WHO goals – In a moderate transmission setting, a vaccine that protects for 5 years can achieve the WHO goals – In a high transmission setting, cohort vaccination can achieve only the morbidity goal. The best strategy was treating 75% of school-aged children with MDA and vaccinating 1- and 11-year-olds –Community-wide immunization has the greatest impact on the burden of heavy intensity infection in the community and offers the greatest chance of achieving the WHO goals

**Table 8:** Overview of modelling studies that measure anticipated socio-economic impact of the vaccine.

Policy question	Assessment method/measure	Additional information specific to models	Assumptions	Outcomes/interpretation

Investigate the cost-effectiveness of a potential vaccine and calculate a critical vaccination cost [114]	–Cost-effectiveness measured as cost per high intensity infection years averted over 30-year period	–Stochastic individual-based transmission model with *S. mansoni* explicitly transmitted between human hosts and external reservoir –Matched to data from epidemiological studies in Kenya	–Vaccination cost US$3, $6, and $12 per full course, including delivery –Health care provider perspective –Vaccine reduces establishment of new worms by 90% and reduces fecundity by 90%	–In high transmission settings, when vaccines confer <20 years protection, vaccinating school aged children every 5 years is most cost-effective, if protection lasts 20 years, immunizing children in cohorts is most cost-effective. Vaccination could cost up to US$8 and remain more cost-effective than MDA (i.e., critical vaccination cost) –In moderate transmission settings, only when long-lasting and cost less than $3.7 was vaccination more cost-effective compared to MDA –Cost-effectiveness is highly dependent on transmission setting, duration of vaccination protection, and cost of vaccination
Is a vaccine alone or in combination with a drug treatment a more effective strategy to achieve the World Health Organization 2020–2025 goals? [115]	–Cost-effectiveness measured as cost per high intensity infection years averted over 30-year period	–Used two different models –Both individual-based transmission models –Cost-effectiveness calculated as high intensity infection years averted per US $ spend over course of the intervention	–Vaccine used either alone or in combination with MDA –Vaccine reduces rate of infection and rate of egg production by 100% –Vaccination cost was for all doses and included cost of the vaccine, supply chain, and service delivery	–In high transmission settings, MDA is more cost-effective when vaccine cost US$12, if vaccine cost *<*US$6, vaccinating school-aged children every 5 years was most cost-effective for all durations of protection (5–20 years). This strategy is most cost-effective up to US$9.20 –In low to moderate transmission settings, MDA more cost-effective when vaccine cost ≥US$6, when it cost US$3, vaccination of school-aged children every 5 years most cost-effective –Cost-effectiveness depended on transmission setting, duration of vaccine protection, level of coverage, and cost of treatment
Investigates the characteristics of the vaccine that would be required in order to be more cost-effective than chemotherapy [119]	–Cost-effectiveness	–Effectiveness measured in number of years of heavy infection prevented or cured –Cost model/calculation	–Focuses on school aged children in high transmission areas –No immediate impact of vaccine on transmission –Provider perspective, includes cost vaccine and delivery –5% discount rate	–If vaccine offers protection for 15 years and is introduced into the EPI program, vaccination dominates (i.e., less costly and more effective) chemotherapy as long as additional cost of adding vaccine to EPI is less than $5.33 (assumes 80% coverage, 90% compliance) –Vaccination would be more cost-effective than chemotherapy if offering at least 6 years of protection –Vaccination of all 6–15-year-olds administered every 2–6 years would be cost-effective compared to chemotherapy when the cost of vaccination is ≤$5.80
Estimate the costs of severe stages for *S. mansoni* in Brazil in 2010 [120]	–Cost-of-illness	–Included costs associated with hospitalization and diagnosis, treatment, and meals during the hospital stay as well as care prior to admission and days missed from work due to hospitalization and post-discharge recovery –Potential life years lost due to premature death used to estimate indirect costs due to mortality –Sensitivity analysis varied cost estimates	–Population was those hospitalized for *S. mansoni* as per reported to the Ministry of Health (410 individuals) –Length of stay based on current patient population –Assumptions for lost productivity in line with Brazil’s labor regulations & wages with social security discounts	–Total cost was US$20.4 million –Costs to public health sector totalled US$0.5 million –Costs of productivity losses totalled US$19.9 million –96% of costs were related to lost productivity
Estimate the economic burden of *S. mansoni* in Brazil in 2015 [121]	–Cost-of-illness based on prevalence approach from a societal perspective	–Included direct health care costs (e.g., stool samples, diagnosis, hospitalizations), indirect costs (e.g., transportation and lost wages due to death and missed work) –Potential life years lost due to premature death used to estimate indirect costs due to mortality –Sensitivity analysis varied number of cases and severity level and cost estimates	–Costs estimated for 26,499 carriers –Cost estimates for transportation and caregivers determined from interviews –Uses human capital approach	–Total cost US$41.7 million –Total direct costs US$2.2 million –Total indirect costs US$39.5 million –94.6% of costs were indirect costs

**Table 9:** Overview of expectations of evidence that are likely to be required to support a global/regional/national policy recommendation, or financing.

Parameter for policy/financing consideration	Detailed parameters	Assumptions	Guidance/reports available

Health impact	– Total future deaths averted in the 2020–2035 period, and per 100,000 vaccinated – Total future cases averted in the 2020–2035 period, and per 100,000 vaccinated	N/A	[123]
Value for money	– Vaccine procurement cost per death averted – Vaccine procurement cost per case averted	N/A	[123]
Equity and social protection impact	– Disproportionate impact of disease on vulnerable groups – Special benefits of vaccination for women and girls	N/A	[123]
Economic impact	– Direct medical costs averted – Indirect costs averted	N/A	[123]
Global health security impact	– Epidemic potential of disease – Impact of vaccination on antimicrobial resistance (AMR)	N/A	[123]
Other impact	– Total under-five deaths averted in the 2020–2035 period, and per 100,000 vaccinated – Total DALYs averted in the 2020–2035 period, and per 100,000 vaccinated – Vaccine procurement cost per disability-adjusted life year (DALY) averted	N/A	[123]
Gavi comparative advantage	– Degree of vaccine market challenges – Potential for Gavi support to catalyse additional investment	N/A	[123]
Implementation feasibility	– Ease of supply chain integration – Need for healthcare worker behaviour change – Feasibility of vaccination time point – Acceptability in target population – Long-term financial implications	N/A	[123]
Alternative interventions	– Optimal use of current and future alternative interventions (prevention and treatment)	Urinary and intestinal schistosomiasis can be treated with Praziquantel (PZQ) for 1–2 days [124].	[123]
Broader health system benefits	– No specific indicator – evaluated on a case-by-case basis	N/A	[123]
Vaccine cost	– Total procurement cost to Gavi and countries, 2020–2035	N/A	[123]
Operational cost	– Incremental in-country operational costs per vaccinated person	N/A	[123]
Additional implementation costs	– Additional costs for introduction	N/A	[123]

## Data Availability

Review of publicly available data

## Appendix A. Framework to inform section 5 – vaccine development

**Table:**

Themes Considered	Indicators	Very Low	Low	Moderate	High	Very High

**Biological Feasibility**	Most advanced vaccine candidate (s)	• Ph1, or preclinical, or no candidates in the pipeline	• Ph2 candidate	• Ph3 candidate	• Ph3 candidate with high likelihood of licensure by a WHO-listed national regulatory authority	• Licensed vaccine by a WHO-listed national regulatory authority
**Biological Feasibility**	Existence of immunity from natural exposure	• No evidence that natural exposure confers immunity	• Conflicting or minimum evidence	• Some evidence and/or immunity of limited duration	• Good evidence of relatively long-lasting immunity	• Well established that natural exposure confers protects against severe disease (or indicated vaccine outcome) with durable immunity
**Biological Feasibility**	Understanding mechanisms of immunity	• Mechanisms of pathogen induced immunity unknown	• Very limited or conflicted understanding of pathogen-induced immunity and/or immune-enhanced disease	• Some understanding of pathogen- induced immunity and whether immune-enhanced disease exists	• Good understanding of pathogen- induced immunity, however some mechanisms remain unclear; evidence that immune-enhanced disease is unlikely	• All mechanisms of pathogen-induced immunity are well understood; robust evidence that immune-enhanced disease is rare
**Biological Feasibility**	Likelihood of vaccine protection against the majority of pathogenic strains	• Evidence that a vaccine would not protect against majority of pathogenic strains, or gap in evidence that a vaccine would protect against majority of pathogenic strains	• Limited evidence that a vaccine would protect against majority of pathogenic strains	• Some evidence that a vaccine would protect against majority of pathogenic strains	• Strong evidence that a vaccine would protect against majority of pathogenic strains	• No known strain variation that is relevant to a vaccine OR evidence that a vaccine will protect against all known strains OR new strain can be rapidly developed
**Product Development Feasibility**	Existence of animal models to facilitate vaccine development	• Animal models do not exist and no progress has been made to identify them.	• Animal models do not exist but some progress has been made to identify them.	• Animal models are identified but their utility have not been confirmed	• Animal models are identified and used but the mechanisms of immunity are unclear	• Animal models are well–defined and used and mechanisms of immunity are well understood OR animal models are not required for vaccine development
**Product Development Feasibility**	Existence of in vitro assays to facilitate vaccine development	• In vitro assays do not exist and no progress has been made to develop them.	• In vitro assays do not exist but some progress has been made to develop them.	• In vitro assays are developed but their utility has not been established or the assays have not been analytically qualified	• In vitro assays are analytically qualified and used but their fit for purpose as a relevant biomarker for decision-making or licensure has not been established	• In vitro assays are qualified/validate and are fit-for-purpose for decision-making or licensure
**Product Development Feasibility**	Ease of clinical development	• A complex trial design (no correlate) and a significant investment in clinical sites/infrastructure needed for testing a vaccine OR low disease incidence a significant impediment to efficacy trial feasibility	• A complex trial design needed (no correlate) that may require investment in clinical sites/infrastructure OR low disease incidence requires a large and/or long efficacy trial	• A standard trial design that may require investment in clinical sites/infrastructure	• Common trial design, potential correlate and a possibility to leverage existing trial infrastructure.	• Common trial design, established correlate and a possibility to leverage existing trial infrastructure AND high disease incidence allows for an efficacy trial.
**Product Development Feasibility**	Availability or need for human challenge models	• Human challenge models OR diagnostic tools are important for vaccine development but are not developed.	• Human challenge models OR diagnostic tools are important for vaccine development and some progress has been made to develop them.	• Human challenge models OR diagnostic tools are important for vaccine development, are developed but not used or their use is unclear.	• Human challenge models OR diagnostic tools would facilitate vaccine development and are developed but their use is limited.	• Human challenge models OR diagnostic tools are important for vaccine development, are developed and widely used and accepted, OR no human challenge model is required

## Appendix B. Framework to inform section 9 – access and implementation feasibility

**Table:**

Themes Considered	Indicators	Very Low	Low	Moderate	High	Very High

**Access and Implementation Feasibility**	Possibility of implementation within existing delivery systems	• No possibility to leverage existing delivery systems due to a complex vaccine immunisation schedule	• Some evidence that existing delivery systems could be leveraged to deliver a vaccine	• Limited use of existing delivery systems to deliver a vaccine	• Vaccine can be delivered within existing delivery systems with amendments	• Vaccine can be delivered within existing delivery systems as is
**Access and Implementation Feasibility**	Commercial attractiveness	• Poorly defined target population • Disease burden mainly in LMICs but vaccine unlikely to be supported by Gavi	• Small target population predominantly in LMIC public markets • Difficulty defining target population in LMICs	• Large target population distributed predominantly in LMICs with potential Gavi support	• Well-defined target population in LMIC public markets • Large target populations distributed across HIC and LMIC markets	• Large target population in HICs and LMICs, both private and public markets
**Access and Implementation Feasibility**	Clarity of licensure and policy decision pathway	• A need for novel licensure and/or policy pathway, which is currently unclear	• A need for novel licensure and/or policy pathway	• A possibility to leverage an existing licensure and policy pathway with major amendments	• A clear licensure and policy pathway with minor amendments	• A clear, highly precedented, fit for purpose licensure and policy pathway currently exists
**Access and Implementation Feasibility**	Expected financing mechanism	No interest from global funders or national procurement agencies, potential for private market	Unlikely to be of interest to global funders, requiring commitment from national procurement	Potential interest from global funders, depending on public health impact data, interest from national procurement agencies	High level of interest expressed from public financing agencies such as Gavi, PAHO RF, and from national procurement agencies	Advanced purchasing commitment from, for example Gavi, PAHO RF, or other pull mechanism(s) in place
**Access and Implementation Feasibility**	Ease of uptake	• Extensive challenges with a new vaccination touchpoint required • High level of clinician judgement and clinical engagement • Additional extensive barriers to uptake including lack of national commitment	• Evidence of low uptake for marketed vaccines • Cultural barriers, negative patient perceptions	• New vaccination touchpoint required	• Well-defined target population with likelihood of high acceptability, but possible difficulties in infrastructure for vaccination	• Well-defined target population with likelihood of high acceptability • Evidence of high uptake for marketed vaccine • Lack of other significant barriers to introduce a vaccine • Strong national commitment to introduce a vaccine
